# Targeting conformational changes in C‐reactive protein to inhibit pro‐inflammatory actions

**DOI:** 10.15252/emmm.202217003

**Published:** 2022-12-05

**Authors:** János G Filep

**Affiliations:** ^1^ Department of Pathology and Cell Biology University of Montreal Montreal QC Canada; ^2^ Research Center Maisonneuve‐Rosemont Hospital Montreal QC Canada

**Keywords:** Immunology, Microbiology, Virology & Host Pathogen Interaction, Pharmacology & Drug Discovery

## Abstract

C‐reactive protein (CRP) is a marker of acute inflammation and modulator of host defense against infections. CRP exists in conformationally distinct forms that exhibit opposing biological functions and could amplify tissue damage. Therefore, therapies that efficiently target the deleterious actions of CRP are needed. In this issue of *EMBO Molecular Medicine*, Zeller *et al* report development of a novel low molecular weight phosphocholine‐mimetic that binds to pCRP and inhibits conformation change‐mediated expression of pro‐inflammatory actions without impairing its defense function and demonstrate its beneficial actions in preventing rejection of allograft transplants and renal ischemia–reperfusion injury.

## Graphical abstract

J. Filep discusses the recent study from Zeller *et al* (in this issue of *EMBO Mol Med*) that reports the development of a low molecular weight phosphocholine‐mimetic that binds the pentameric C‐reactive protein and inhibits CRP pro‐inflammatory effects without impairing its defense function.

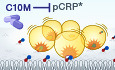

The acute‐phase reactant pentameric C‐reactive protein (pCRP) is a biomarker of inflammation and an essential component of host defense. There is evidence that administration of human pCRP could aggravate tissue injury in animal models (Pepys *et al*, 2006; McFadyen *et al*, 2018). Clinical studies reported correlation between elevated plasma CRP levels with myocardial infarct size (Ries *et al*, 2021) or the severity of COVID‐19‐evoked lung injury (Torzewski *et al*, 2022). CRP exists in conformationally distinct forms and dissociation of pCRP into its subunits monomeric CRP (mCRP) results in expression of potent pro‐inflammatory properties (Wu *et al*, 2015). The Eisenhardt and Peter laboratories identified that binding of pCRP to phosphocholine (PC) or phosphoethanolamine (PE) head groups of membrane lipids, exposed on the surface of activated platelets or damaged cells, induces changes in the pentameric conformation, yielding a partially dissociated pentamer (pCRP*), which then dissociates into the monomeric subunits (Eisenhardt *et al*, 2009; Braig *et al*, 2014). Deposition of pro‐inflammatory CRP isoforms in inflamed/injured but not in healthy tissues, and tissue expression of mCRP would ensure localization of inflammation (Eisenhardt *et al*, 2009) and precipitate tissue injury (Wu *et al*, 2015). The concept of activation‐induced conformational changes could explain why pCRP itself is not pro‐inflammatory in the absence of tissue injury or infection. Therefore, targeting the mechanisms that trigger conformational changes in pCRP and unmask “hidden” pro‐inflammatory activities is of therapeutic interest.

Several CRP targeting strategies have been developed. In 2006, the Pepys group developed bivalent compounds, such as 1,6‐bis(phosphocholine)‐hexane (bis‐PC) that bridges the PC‐binding pockets on the B‐face of two separate CRP pentamers, bringing the PC‐binding surfaces (i.e., the B‐face) together in a parallel fashion (Pepys *et al*, 2006). The resulting decamer structure prevents conformational changes in pCRP and the binding of other ligands to the B‐face. Other CRP targeting strategies include peptide mimetics, anti‐sense oligonucleotides (Warren *et al*, 2015) or reducing serum pCRP levels via CRP‐apheresis using PC‐linked resins (Ries *et al*, 2021). These approaches are currently being investigated in clinical trials with promising initial results reported for reducing tissue injury in patients with myocardial infarction (Ries *et al*, 2021) or COVID‐19‐evoked respiratory distress (Torzewski *et al*, 2022). Arguably, therapies aimed at reducing serum pCRP level could potentially impair antibacterial defenses. Thus, an attractive alternative approach is to selectively block expression of pro‐inflammatory properties “hidden” in pCRP.

In their present study, Zeller *et al* (2022) employed a combination of medicinal chemistry and computational modeling to develop a low molecular weight monovalent tool compound C10M [3‐(dibutyl amino) propyl) phosphonic acid]. Using X‐ray crystallography, the authors demonstrate that C10M binds to the PC/PE binding pocket on pCRP and competitively inhibits the binding of pCRP to exposed PC/PE head groups on bioactive lipids and subsequently prevent formation of pCRP*/mCRP (Fig 1). The monovalent compound allows the B‐face of pCRP, apart from the occupied PC/PE binding pocket, to be accessible to other interacting ligands, including binding of misfolded or aggregated proteins or proteins whose secondary structure is predominantly β‐sheet (Hammond *et al*, 2010) as well as neutrophils.

**Figure 1:**
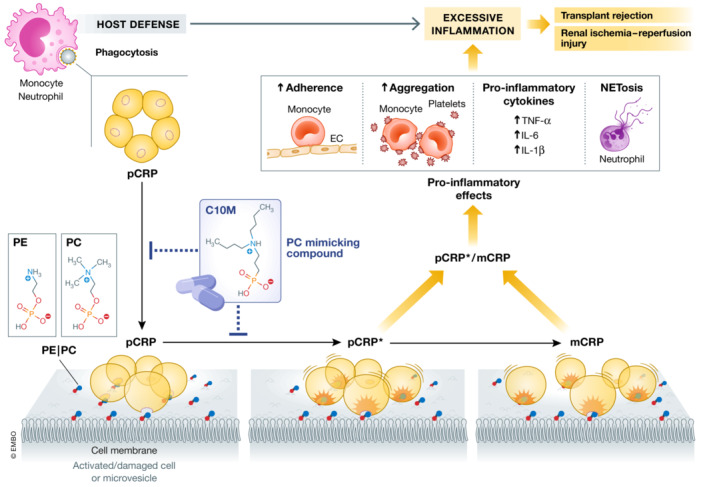
Targeting pro‐inflammatory conformational change in C‐reactive protein Disc‐shaped pentameric CRP (pCRP) functions as an opsonin to enhance phagocytosis of bacteria by monocytes and neutrophils. Binding of pCRP to phosphocholine (PC) or phosphoethanolamine (PE) head groups exposed on the surface of activated or damaged cells, or microvesicles containing PC head groups, leads to conformational changes, yielding a partially dissociated pentamer (pCRP*) followed by dissociation into its monomeric subunits (mCRP). Generation of pCRP*/mCRP results in expression of pro‐inflammatory actions, including stimulation of monocyte adhesion to endothelial cells, enhanced formation of platelet–monocyte aggregates, production of pro‐inflammatory cytokines TNF‐α, IL‐1β, and IL‐6, and triggering extrusion of neutrophil extracellular traps (NET). These contribute to rejection of hindlimb allografts and renal ischemia–reperfusion injury in rats. The monovalent CRP inhibitor C10M prevents binding of pCRP to PC/PE and conformational change in pCRP toward pCRP*/mCRP and consequently reduces pro‐inflammatory actions. These would result in protection against transplant rejection and ischemia–reperfusion injury without affecting pCRP‐mediated phagocytosis.

Consistent with inhibition of pCRP binding and the conformational change to pCRP*/mCRP, the authors document the potency of C10M to inhibit pCRP*/mCRP‐stimulated activation of monocytes and neutrophils (assessed by CD11b expression and generation of reactive oxygen species), adhesion of monocytes to fibrinogen matrix and activated endothelial cells, and formation of platelet–monocyte aggregates. C10M markedly reduced expression of ICAM‐1 and VCAM‐1 on human endothelial cells challenged with pCRP* expressed on platelets or microvesicles, indicating modulation of a mechanism crucial in transporting and mediating pCRP* in the blood *in vivo*. Furthermore, C10M reduced production of pro‐inflammatory cytokines TNF‐α, IL‐1β, and IL‐6 by monocytes. Of note, pCRP*/mCRP triggered the extrusion of neutrophil extracellular traps (NET), presumably the suicidal form, and this was also attenuated by C10M. Reducing suicidal NET formation is of particular relevance because of the potential role of NET in mediating tissue damage. Importantly, C10M did not affect pCRP opsonization‐mediated phagocytosis of bacteria by monocytes and neutrophils, indicating preservation of antibacterial host defense (Fig 1).

To further assess the therapeutic potential of C10M against pCRP‐induced aggravation of tissue injury, the authors tested C10M on murine models of renal ischemia–reperfusion and allograft rejection and found remarkable protection. In both models, C10M markedly suppressed deposition of human pCRP*/mCRP and pCRP‐dependent monocyte accumulation within the affected organs. These were associated with significant improvement of excretory renal function following ischemia–reperfusion, and prevention of premature loss of allograft hindlimb transplants driven by human pCRP.

In summary, Zeller *et al* (2022) provide a proof‐of‐concept of selective targeting the actions of CRP. This work will certainly facilitate future investigations into the full spectrum of pCRP's roles in inflammatory pathologies and opens a novel therapeutic avenue to prevent or limit the harmful actions of pCRP.

## Author contributions

**Janos G Filep:** Conceptualization; formal analysis; funding acquisition; writing – review and editing.
